# Long-term Rescue of Photoreceptors in a Rodent Model of Retinitis Pigmentosa Associated with MERTK Mutation

**DOI:** 10.1038/s41598-018-29631-z

**Published:** 2018-07-27

**Authors:** H. Lorach, S. Kang, R. Dalal, M. B. Bhuckory, Y. Quan, D. Palanker

**Affiliations:** 1 Hansen Experimental Physics Laboratory, Stanford University, Stanford, CA USA; 2 Department of Ophthalmology, Stanford University, Stanford, CA USA; 3 Department of Ophthalmology and Visual Science, College of Medicine, The Catholic University of Korea, Seoul, Republic of Korea

## Abstract

MERTK mutation reduces the ability of retinal pigment epithelial (RPE) cells to phagocytize the photoreceptor outer segments, which leads to accumulation of debris separating photoreceptors from RPE cells, resulting in their degeneration and loss of vision. In a rat model of Retinitis Pigmentosa due to *MERTK* mutation, we demonstrate that surgical removal of debris performed when about half of photoreceptors are lost (P38), allows the remaining photoreceptor cells to renew their outer segments and survive for at least 6 months – 3 times longer than in untreated eyes. In another set of experiments, patterned laser photocoagulation was performed before the debris formation (P19-25) to destroy a fraction of photoreceptors and thereby reduce the phagocytic load of shed outer segment fragments. This treatment also delayed the degeneration of the remaining photoreceptors. Both approaches were assessed functionally and morphologically, using electroretinography, optical coherence tomography, and histology. The long-term preservation of photoreceptors we observed indicates that *MERTK*-related form of inherited retinal degeneration, which has currently no cure, could be amenable to laser therapy or subretinal surgery, to extend the visual function, potentially for life.

## Introduction

Retinitis pigmentosa (RP) is a group of inherited retinal diseases that can lead to profound loss of vision and eventual blindness due to progressive degeneration of photoreceptor cells. These disorders can be caused by a wide variety of genetic defects. To date, nearly 4300 mutations in 79 genes have been reported to cause RP^1^. Many of the associated genes encode proteins that are involved in phototransduction, photoreceptor structure, or photoreceptor gene transcription. MER-proto-oncogene, tyrosine kinase (*MERTK*) gene encodes for a transmembrane protein involved in recognition and phagocytosis of the photoreceptor outer segments, essential for recycling of the phototransduction machinery. Mutations in the *MERTK* gene cause reduced phagocytic function, which leads to accumulation of photoreceptor outer segment debris in subretinal space. This debris subsequently impedes efficient oxygen and nutrient transport to photoreceptor cells. This mutation was actually identified thanks to a spontaneous rodent model of retinal degeneration: the Royal College of Surgeons (RCS) rat. This first animal model of inherited retinal degeneration, was described by Bourne and coworkers in 1938^2^. Since 1970s, it has been known that the RCS rat has dysfunctional RPE cells, which are unable to phagocytize shed outer segment fragments, leading to their accumulation as a debris between photoreceptors and RPE cells^3,4^. Recently, mutations in the *MERTK* gene were identified as the cause of the functional defect in RPE cells of RCS rat^5^. In humans, mutations of the *MERTK* gene lead to a loss of night vision in early childhood, gradual constriction of the visual field, and eventual loss of visual acuity before adulthood. Imaging studies using optical coherence tomography (OCT) in these patients revealed the loss of photoreceptors and formation of a hyper-reflective layer, analogous to the debris layer in RCS rats^6,7^.

In the RCS rats, appearance of the outer segment debris begins around postnatal day (P) 19. These debris accumulate with age^8^, forming a thick insulating layer between photoreceptors and RPE cells by P35, accompanied by gradual degeneration of photoreceptors, which is complete by P180. RCS animal model is widely used today in research of retinal degeneration, including strategies for RPE cell transplantation^9–14^, gene therapy^15–18^ or retinal prosthetics^19–23^. Many of these studies do not introduce a proper sham procedure group, while a few that do, often report a protective effect in the sham surgery group, but tend to undermine and qualify it as a short-term effect^24^. One study^25^, focused on the photoreceptors rescue effect by retinal detachment, demonstrated anatomical rescue comparable to results with RPE transplantation. Unfortunately, this study followed the animals only for 2 months and was not designed to show functional preservation of vision.

Since retinal outer segments are shed daily, but debris accumulation does not start for a few years in patients and for a few weeks in RCS rats, while retina continues to function long after that, we hypothesized that the mutant RPE cells retain some phagocytic activity either by *MERKT*-independent uptake or microglia-related phagocytosis^26^, and thereby can sustain a fraction of the outer-segment recycling load^27^. We also assumed that photoreceptors degeneration is accelerated by accumulation of a thick debris layer, which prevents oxygen and nutrients supply from the choroid. Based on these hypotheses, we designed two strategies to balance the supply and demand of the outer segment recycling and thereby extend the survival of photoreceptors in RCS rats.

Both strategies utilize clinically readily available techniques. First, debris layer can be surgically washed out from subretinal space when a substantial fraction of functional photoreceptors is still present. Alternatively, pattern laser photocoagulation can be applied to selectively destroy a fraction of photoreceptors prior to debris formation. In both approaches, the remaining photoreceptors could be better sustained by the partially functioning RPE cells and survive much longer. In this study, we assess efficacy of these therapies for protection of photoreceptors in RCS rats as a potential therapy for patients with RP due to *MERTK* mutation.

## Results

### Clearance of the outer segment debris by subretinal lavage

**Figure 1:**
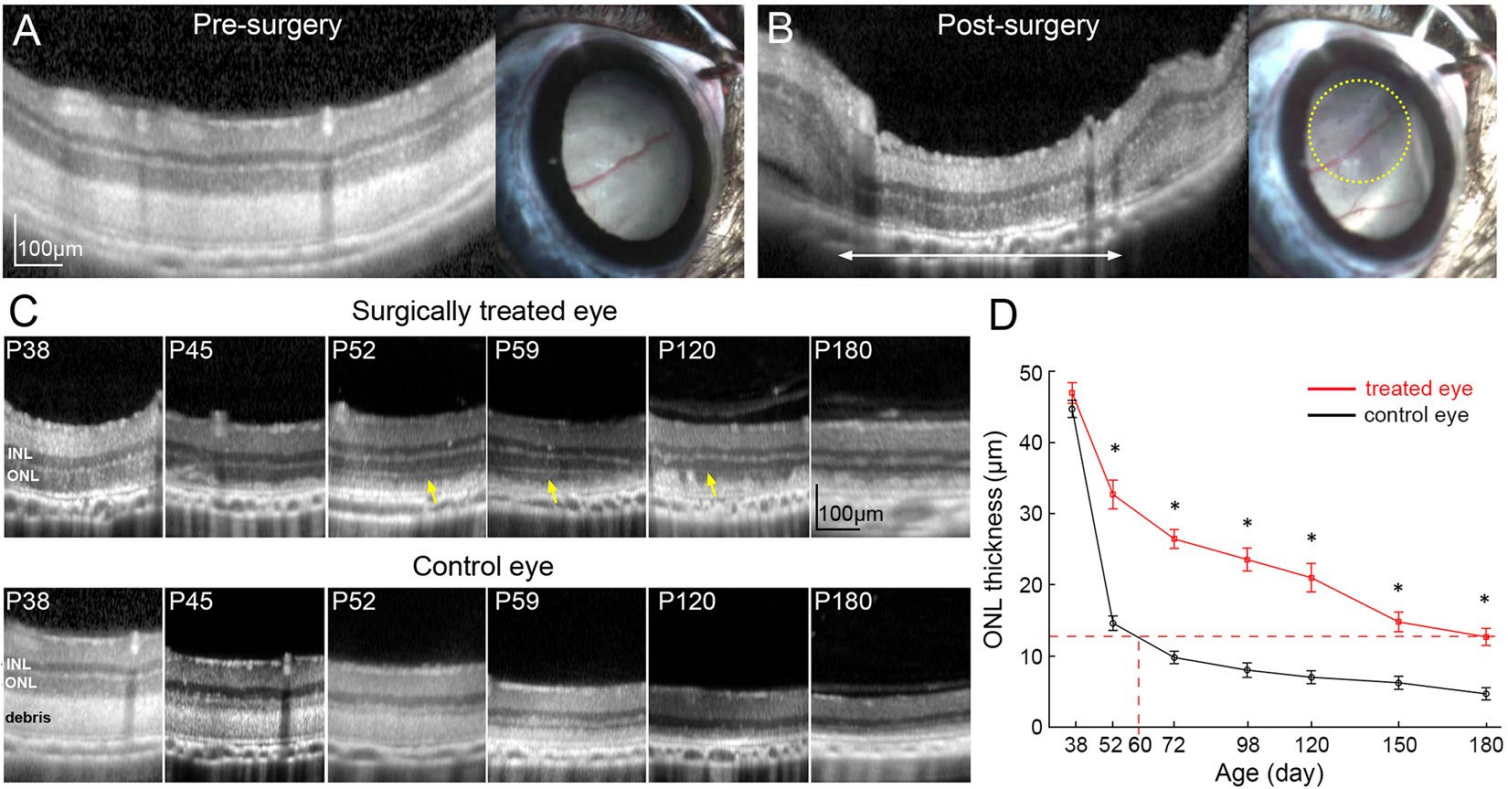
Removal of subretinal debris in RCS rats preserves photoreceptors. (**A**) OCT and fundus image before surgery. (**B**) After the surgery, debris-free area in OCT image is outlined with a white arrow. Retina became more transparent on fundus photography (within the yellow dotted line). (**C**) Serial OCT scans demonstrate better preservation of photoreceptors in treated eyes, as compared to control eyes. Photoreceptor outer segments reappear a few days after debridement (*yellow arrows*). (**D**) ONL is significantly thicker in the treated area than in the corresponding controls. In treated eyes, the ONL thickness at P180 is equivalent to that of P60 in controls (n = 10 control eyes and 10 treated eyes in 10 animals, error bars - s.e.m., *p < 0.01, two-sided paired t-test, p-values respectively 0.36, 2.5E-05, 8.2E-07, 2.2E-05, 0.00023, 0.0014, 5.8E-05).

Retinas in RCS rats develop normally and appear healthy under OCT examination until P19. After that time, the outer segments start accumulating in the subretinal space and form a thick layer by P38, accompanied by thinning of the outer nuclear layer (Fig. 1). RCS rats (n = 14) were treated at P38, when more than half of the outer nuclear layer is still present. The subretinal surgery consisted of a transcleral incision and retinotomy of 1 mm, allowing for insertion of a 30G blunt cannula in the subretinal space (see Methods and Suppl. Video 1). By gentle scraping and saline injection, the thick debris layer was removed in about 2 × 2 mm area, which brought the outer nuclear layer down to the RPE layer (Fig. 1A,B). No subretinal bleeding was observed during surgery. The relatively large sclerotomy allowed fluid and debris extrusion, as opposed to a small single puncture typically used for retinal detachment.

### Long term preservation of the outer retinal morphology and function after subretinal lavage

Natural process of degeneration, observed in untreated eyes, begins with formation of a debris layer insulating the photoreceptors from the RPE and choroid (Fig. 1C). Upon gradual thinning of the outer nuclear layer (ONL), the debris layer is also progressively reduced. By P120, the ONL is not discernable and the inner retina lies close to the RPE.

In contrast, in the treated eyes, we observed a local regrowth of the outer segments in the region with removed debris as early as 2 weeks after surgery (Fig. 1C, arrows) and preservation of the ONL. Debris started increasing locally around P120, correlated with a decrease in ONL thickness. OCT measurements (see Methods) demonstrated significantly thicker ONL in treated eyes, until the end of the follow-up period at 6 months (Fig. 1D). In treated eyes, the ONL thickness at P180 corresponded to that of P60 in the untreated controls, which can be interpreted as extension of the photoreceptors longevity by a at least a factor of 3.

**Figure 2:**
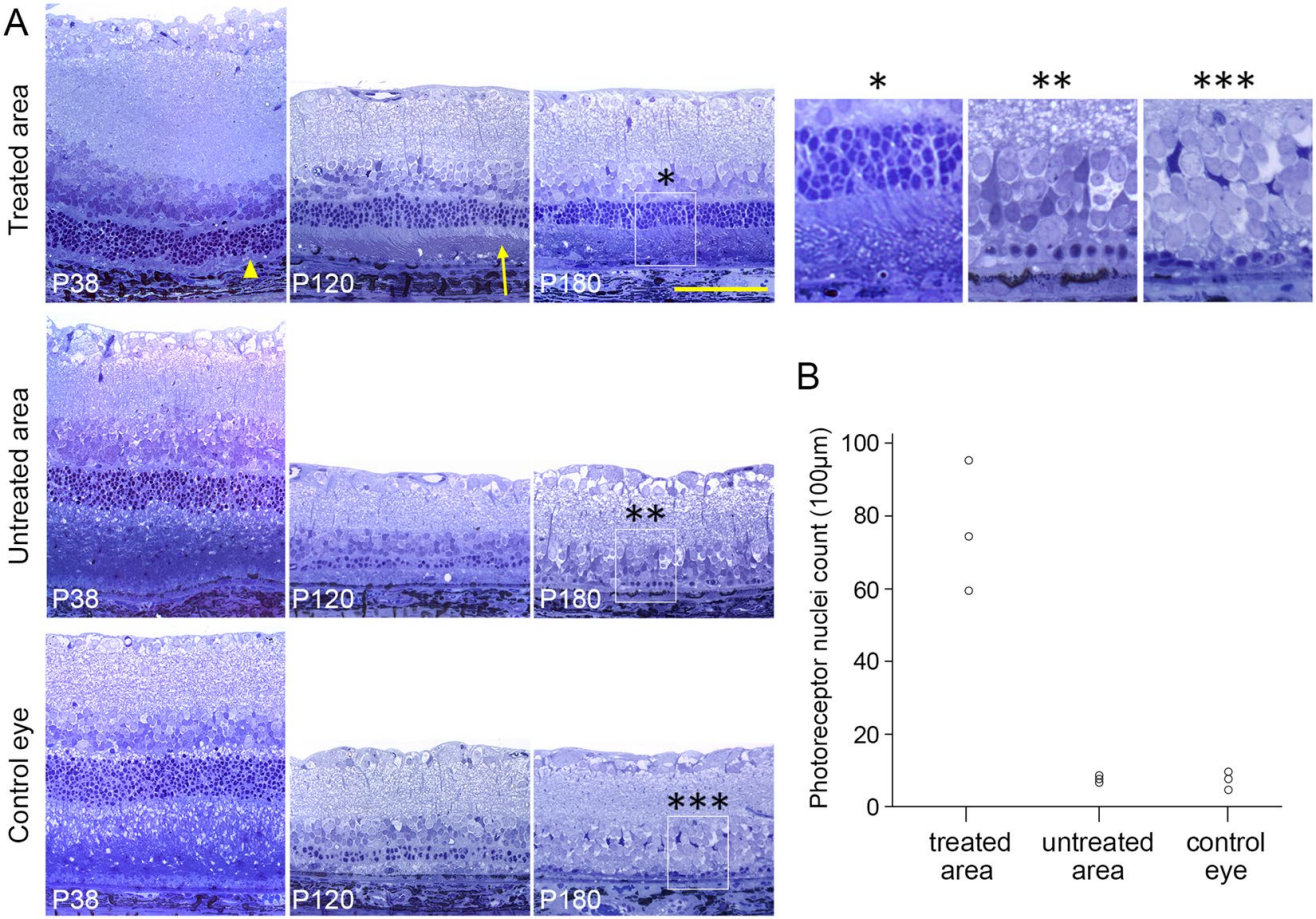
Histology of surgically treated and control eyes. (**A**) *Upper row:* histology of the 38, 120, and 180-day old RCS rats in the surgically-treated area. *Middle row:* untreated retina in the same eye. *Bottom row:* and contralateral control eye. Treated retina exhibited much thicker ONL than both controls. Note that after subretinal lavage, subretinal debris was removed (P38, *arrow head*) and outer-segments reappear (P120, *arrow*). (**B**) Number of the photoreceptor nuclei per 100 µm in the surgically treated retina, untreated retina, and control eye (n = 3 control eyes and 3 treated eyes in 3 animals).

Histological analysis (P38, P120 and P180) confirmed much better preservation of ONL and maintenance of the outer segment morphology for up to 6 months in the treated eyes (Fig. 2). Transmission electron microscopy confirmed the presence of the inner and outer segments, as well as phagosomes in the RPE (Suppl. Fig. S1). At P180, as much as 4–6 rows of photoreceptor nuclei were observed, as well as inner and outer segments, whereas the untreated area of the same eye and control eyes displayed at most 1 layer of nuclei, with no discernable outer and inner segment structures. At P180, in the treated region, we observed preservation of 76 ± 10 (s.e.m.) nuclei per 100 µm, compared to 7 ± 0.6 in untreated area and 6.7 ± 1.5 in the fellow eyes (Fig. 2B). Immunostaining against rhodopsin confirmed the health of the outer segments. Cone arrestin staining demonstrated presence of some cones, with their distinct outer segment structures and cone pedicles, in this rod-dominated rat retina (Suppl. Fig. S2). Immunostaining against GFAP showed similar activation of Müller cells in the treated and control areas, with no correlation with the rescue of photoreceptors (Suppl. Fig. S3B). Microglial cells detected with IBA1 immunoreactivity were found throughout the retina, including the outer segments area (Suppl. Fig. S3A). Overall, the volume covered by glial cells in the outer segment zone was minor. These results confirm that, even though the treated retina is in a reactive state, with upregulated GFAP and migrated microglia, the structure of photoreceptors is still preserved, in agreement with functional measures described below.

**Figure 3:**
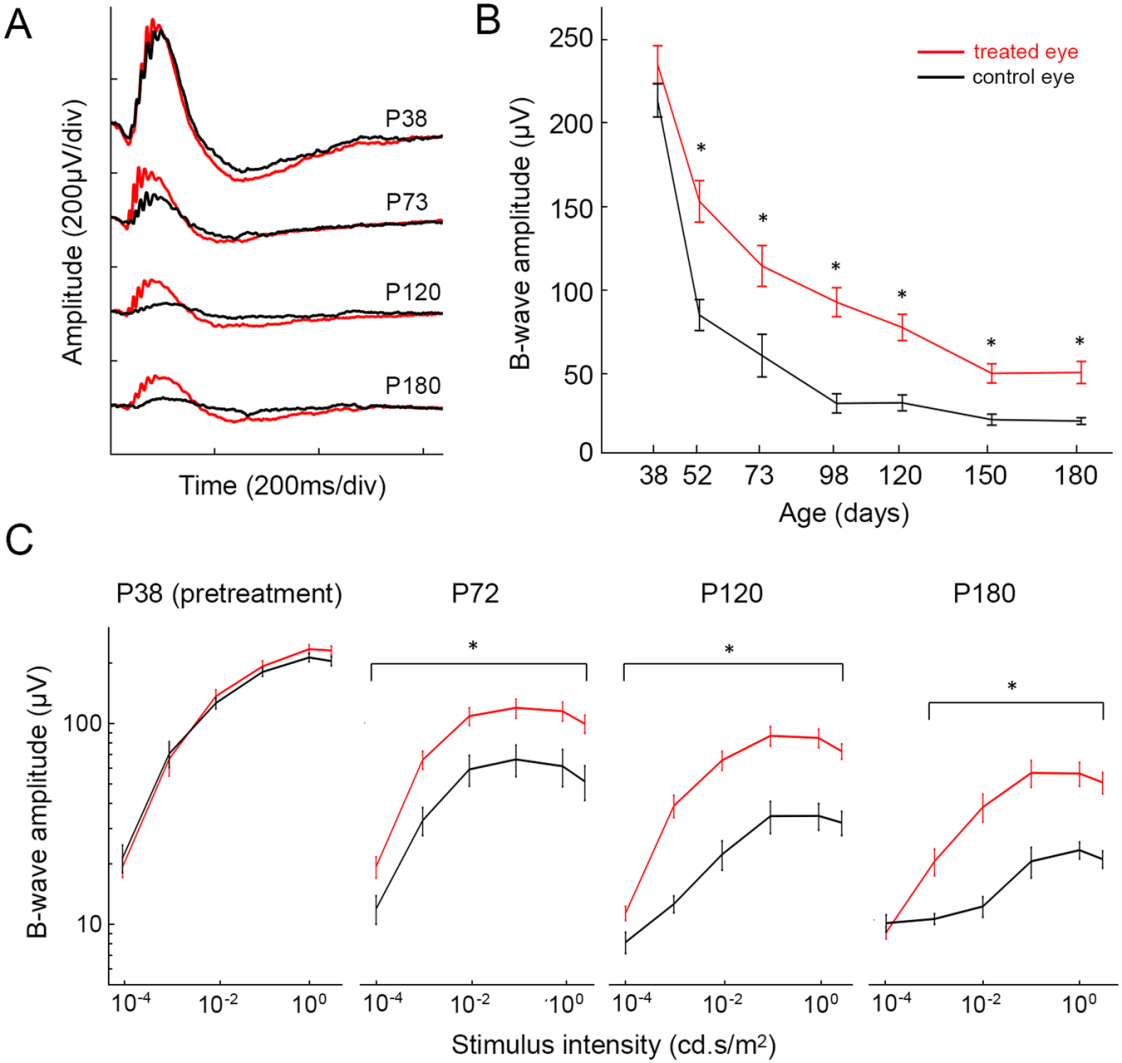
ERG response in surgically-treated and a control eye. (**A**) Representative ERG waveforms from 38, 73, 120, and 180-day old RCS rats. (**B**) B-wave amplitudes as a function of age show significant difference between the treated and control eyes. Stimulus intensity was 1 cd.s/m^2^ (n = 10 control eyes and 10 treated eyes in 10 animals, error bars - s.e.m., *p < 0.05, two-sided paired t-test, p-values for each time point: 0.11, 9.9E-05, 0.012, 1.4E-04, 4.5E-04, 8.4E-05, 5.3E-04). (**C**) B-wave amplitude (log scale) with stimuli ranging from 10^−4^ to 3cd.s/m^2^. (n = 10 control eyes and 10 treated eyes in 10 animals, error bars - s.e.m., *p < 0.05, two-sided paired t-test).

For functional assessment, we measured dark-adapted full-field flash electroretinograms (ERG) at various light intensities. In both the treated and untreated eyes, the ERG response decreased with age (Fig. 3A). However, treated eyes exhibited significantly (p < 0.05, paired t-test) higher b-wave amplitude at all time points, starting 2 weeks post-surgery (Fig. 3B). Since morphological preservation of photoreceptors was observed only in the treated areas, the rest of the retina still undergoes degeneration, and the difference between the two curves illustrates the contribution from the treated area, which is seen until the end of the 6-months follow up period.

### Pattern laser photocoagulation to reduce photoreceptors density

**Figure 4:**
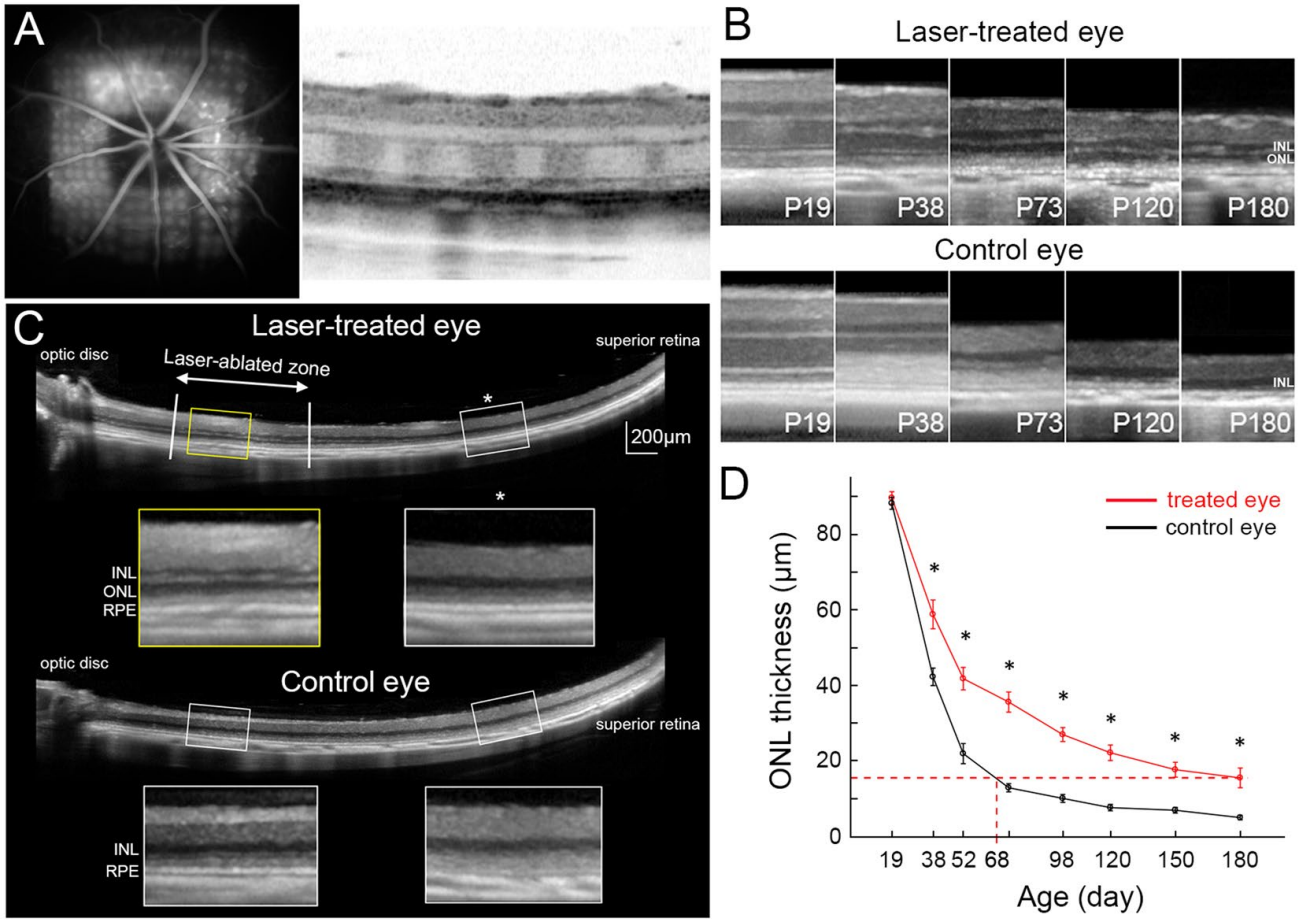
Preservation of retinal structure after laser photocoagulation. (**A**) Laser was applied to the central retina around the optic nerve head at P19. Pattern of photocoagulation can be seen in fluorescein angiography and in OCT. (**B**) Laser treatment delays the loss of photoreceptor nuclei from P19 through P180, compared to control eyes. (**C**) ONL was much thicker in laser-treated retina (*yellow box*) compared to untreated periphery (*white box with asterisk*). Nearly no ONL could be seen in the corresponding zones of the control eye (*lower two white boxes*) (P98). (**D**) Significantly higher ONL thickness in the laser-treated retina, compared to the corresponding areas of the control eye. In treated eyes, the ONL thickness at P180 is equivalent to that of P68 in control eyes. (n = 8 control eyes and 8 treated eyes in 8 animals, error bars - s.e.m., *p < 0.05, two-sided paired t-test, p-values respectively 0.29, 4.7E-04, 7.1E-04, 1.2E-05, 8.3E-06, 5.6E-04, 8.1E-04, 0.011).

The second approach was based on reducing the recycling load by early (n = 12, P19-P25) photocoagulation of a fraction of photoreceptors. Grid laser patterns with 45 μm spots and 1.5 spot diameter spacing covered about 1.6 × 1.6 mm^2^ in the central retina surrounding the optic disc (Fig. 4 and Methods). Very small laser spots were used to allow redistribution of the remaining photoreceptors into the ablated areas over time, without any scarring^28,29^. The laser coagulation spots appeared hyper-fluorescent on fluorescein angiography (FA) and as light stripes (higher scattering) in the ONL on OCT scan (Fig. 4A). One eye per animal was treated, and the fellow eye served as a control.

### Laser photocoagulation delays the retinal degeneration morphologically and functionally

OCT imaging demonstrated that laser treatment significantly delays the loss of photoreceptors. In the laser-treated eye, the ONL could be identified until P180, while in the untreated fellow eye, photoreceptor nuclei became barely distinguishable after P73 (Fig. 4B). The rescue effect was limited to the treated area, as shown in Fig. 4C. The average thickness of ONL in the laser-treated eyes at P180 was equivalent to that of control eyes at P68 (Fig. 4D) – extension of the longevity by a factor of 2.7.

**Figure 5:**
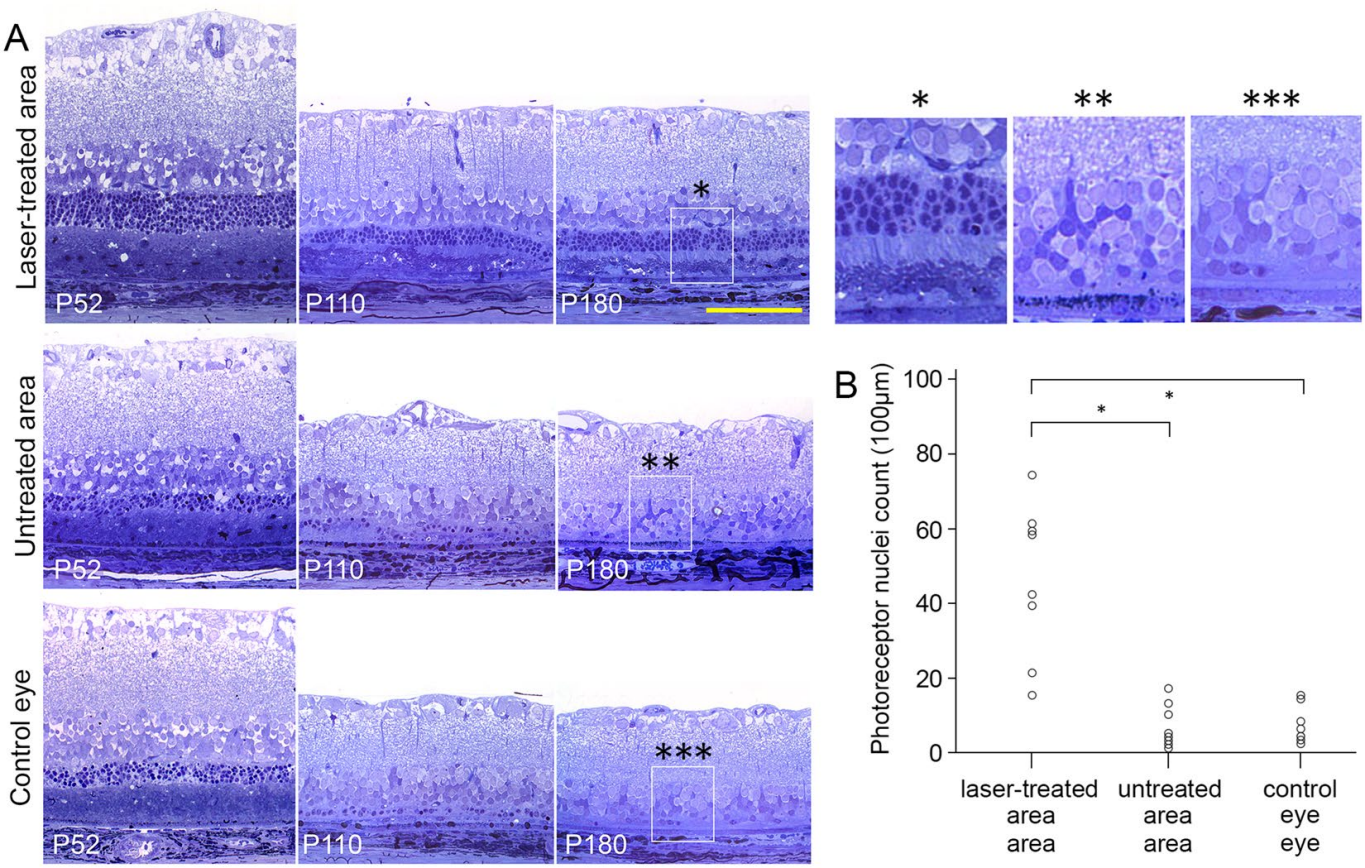
Histology of the laser-treated and control eyes. (**A**) The representative histology of 52, 110, and 180-day old RCS rat retinas from laser-treated area (upper row), untreated retina in the same eye (middle row), and contralateral control eye (bottom row). Much thicker ONL is evident in the treated area than in both controls. (**B**) Number of the photoreceptor nuclei per 100 µm in the laser-treated retina, untreated retina, and control eyes (n = 8 control eyes and 8 treated eyes in 8 animals, error bars - s.e.m; *p < 0.001, two-sided paired t-test).

Histological analysis demonstrated approximately twice thicker ONL in the laser-treated area at P52, compared to untreated area in the same eye or the control eye (*left column* in Fig. 5A). At P110, the treated area had a 3 nuclei-thick ONL, with relatively preserved inner and outer segment structures, while untreated areas and fellow eye displayed, at most, a single row of nuclei with severely truncated and disorganized photoreceptor segments (*middle column* in Fig. 5A). At P180, 3 nuclei-thick ONL and some inner/outer segments were still visible in the treated area, whereas in the controls, most of the photoreceptors disappeared (*right column* in Fig. 5A). The number of nuclei in the laser-treated ONL was significantly higher, compared to both control conditions (Fig. 5B, n = 8, p < 0.05, paired t-test). The average number of nuclei per 100 µm was 45 ± 7.9, 6.9 ± 2.0, and 7.0 ± 1.8 for the laser-treated, untreated retina and control eye, respectively.

**Figure 6:**
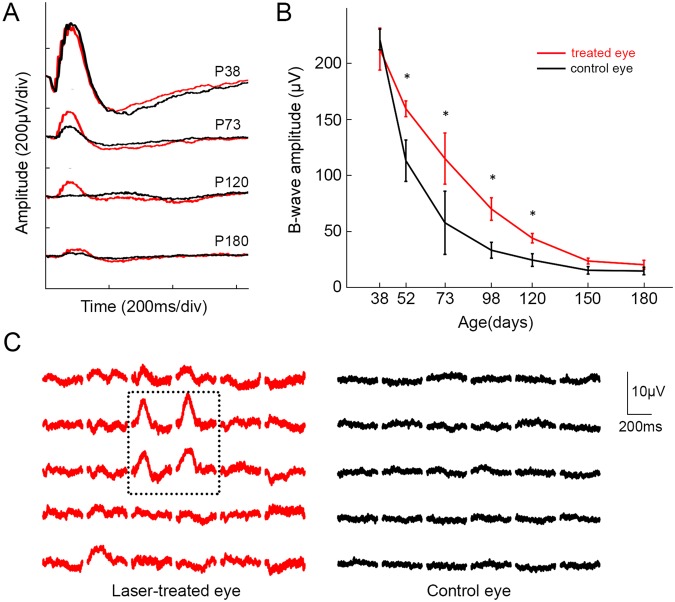
ERG response in laser-treated and control eyes. (**A**) Representative ERG waveforms from 38, 73, 120, and 180-day old RCS rats. (**B**) B-wave amplitude as a function of age. Significant differences are at P52, P73, P98, and P120. Stimulus intensity was 1cd.s/m^2^ (n = 8 animals, error bars s.e.m; *p < 0.05, two-sided paired t-test, p-values 0.91, 0.040, 0.030, 0.011, 0.0046, 0.11, 0.060). (**C**) Topographic distribution of multifocal ERG response in treated (*red*) and control (*black*) eyes (P114). ERG responses within the dotted line correspond to the laser-treated retina. Responses from untreated retina (*red outside dotted line*) or control eye (*black*) were much lower.

Flash-ERG responses in dark-adapted eyes demonstrated a decline with age for both, the laser-treated and control eyes (Fig. 6A). However, b-wave amplitudes in the laser-treated eyes (red lines) were higher than those of the control eyes (black lines) from P38 until the end of the 6 months follow-up period (shown for 1 cd.s/m^2^ in Fig. 6B). The difference was significant (p < 0.05, paired t-test) from P52 through P120. Multifocal ERG (mfERG) demonstrated detectable responses only in the laser-treated area, whereas little to no responses were observed in untreated peripheral retina of the same eye or in the control eye (Fig. 6C). This observation illustrates the local nature of the photoreceptors rescue, and could explain the difference in full field ERGs.

## Discussion

We demonstrated that two relatively simple and readily available treatments, such as subretinal lavage and sparse photocoagulation, could preserve photoreceptors for up to 6 months of age in RCS rats. This corresponds to nearly 3-times longer lifetime, compared to natural course of degeneration. In the case of subretinal lavage, 10 times more photoreceptor nuclei were present at P180 than in control conditions, with a single procedure. These findings have at least two major consequences. First, it provides a readily available treatment option for patients with *MERTK*-associated RP. Second, it calls for proper controls in the studies of the vision restoration based on RCS rats.

While photoreceptor rescue with only subretinal injection of saline has been described as early as 1990^25^, most of the recent studies using RCS rats claim no durable rescue effect in their sham treated groups, or worse - do not even test sham surgeries. These studies include cell transplantation^11,30,31^, retinal prosthetics^19–21^, and most strikingly - gene therapy studies^16–18^ which led to phase I clinical trials^32^ with no sham treatment arm. Interestingly, the clinical study in 6 patients only showed sustained improvement of visual acuity with the lowest dose of the viral injection, and only in one patient.

To the best of our knowledge, the results we obtained with subretinal lavage are equal to - or better than all the reported treatments in RCS rats in terms of the morphological and functional outcomes: dark-adapted b-wave amplitude is significantly higher than in controls at 6 months, and number of photoreceptor nuclei is as high as 6 rows at 6 months. These results are a clear demonstration of the importance of fair controls, including sham surgery.

Like surgical removal of the debris, laser photocoagulation also prolonged photoreceptor survival and preserved better retinal function. The exact mechanism of action in both cases is unknown. We hypothesized that if a significant fraction of photoreceptors is eliminated by either laser photocoagulation or due to degeneration before the debris removal, the load of shed outer segments will decrease, and the RPE cells with reduced phagocytic ability will be able to sustain remaining photoreceptors. This idea of balancing the supply and demand is conceptually similar to panretinal photocoagulation for proliferative diabetic retinopathy, where a significant fraction (about 30%) of photoreceptors in the peripheral retina is destroyed by laser ablation. Reduction in the number of photoreceptors decreases the metabolic load on diseased vasculature and leads to a reduced production of angiogenic factors from hypoxic retina, thereby saving central retina from neovascularization^33,34^. Laser patterns in our study covered only about 5% of the total retinal surface: 2.62 mm^2^ out of 52 mm^2^ ^35^. Since the photoreceptors rescue is limited to the treated area, stronger full-field ERG response should be expected if larger area would be lasered.

Enhanced phagocytosis and associated preservation of photoreceptors could also result from upregulation of the basic fibroblast growth factor (bFGF), which plays a critical role in RPE phagocytosis in RCS rats^36^. This and other factors, such as CNTF, are upregulated following mechanical trauma^37–39^ or photocoagulation^40^, and could contribute to enhancement of phagocytosis and preservation of photoreceptors^36,41^. Even though this upregulation in mRNA levels is only transient, with a decay half-time of at most 10 days for bFGF^37,42^, the protein itself was detected as late as 21 days after laser treatment^43^, suggesting a potential long-term benefit of bFGF for photoreceptor protection. Another possible explanation is the involvement of the infiltrated microglia in the phagocytic process (Suppl. Fig. S3).

Surgical and laser treatments prolonged the lifetime of photoreceptors in RCS rats by about a factor of 3. If similar benefit would translate to human retina, patients may be able to sustain vision nearly life-long, instead of losing their sight in early adulthood. Lasers are available in nearly every ophthalmic clinic, and therefore conventional laser photocoagulation can be tested in RP patients with *MERTK* mutation without major safety concerns. Although the onset of *MERTK*-associated RP occurs in early childhood^7,44^, progression of the disease to the advanced stage typically takes several years, leaving a period of time for clinical evaluation of the treatment efficacy. Since the rescue effect is local, the treatment can be first tested in a peripheral retina, while photoreceptors are still preserved, and retinal structure and function can be easily assessed by visual field test, microperimerty and OCT.

Another laser-based approach to test is a selective ablation of a fraction of RPE cells using microsecond pulses, in order to induce RPE proliferation for potential rejuvenation of their phagocytic capacity. Such treatment does not damage photoreceptors, and RPE cells repopulate the treated area within a few days^45^. If successful, this would provide a non-destructive and non-invasive treatment for RP associated with *MERTK* mutation.

For patients with more advanced degeneration, who already developed subretinal debris, surgical lavage is another easily available option for clinical testing. Its preservation effect in animals is better than much riskier procedures, such as gene therapy or RPE replacement^14,18^.

In conclusion, we demonstrated that surgical removal of subretinal debris and pattern laser photocoagulation in a rat model of RP caused by *MERTK* mutation result in long-term survival of photoreceptors – about 3 times longer than in untreated animals. Both methods can be readily tested and may provide very significant extension of vision in patients with *MERTK*-related RP.

## Materials and Methods

### Animals and experimental design

Pigmented RCS rats were obtained from the Rat Resource and Research Center (RCS-p+/LavRrrc strain, University of Missouri) and housed in standard conditions with 12/12 hour light-dark cycle. A total of 22 RCS rats were used in the experiments. Subretinal lavage was performed at P38 (n = 14, 10 animals followed up to 6 months and 4 for intermediate histology). Laser treatment was performed at P19 (n = 8, 4 followed up to 6 months and 4 for intermediate histology) or P25 (n = 4), when the retina was still transparent. In each experiment, only right eye was treated, whereas the left eye was used as an internal control. Animals were anesthetized with intramuscular injection of 75 mg/kg ketamine and 5 mg/kg xylazine. Corneas were also anesthetized with 0.5% tetracaine hydrochloride, and pupils were dilated with 1% tropicamide and 2.5% phenylephrine ophthalmic solution before each experiment. All procedures were conducted in accordance with the NIH Guide for the Care and Use of Laboratory Animals. All proposed experiments were reviewed and approved in advance by *the Administrative Panel on Laboratory Animal Care Committee at Stanford University*.

### Subretinal lavage

After anesthetizing the animals, subretinal lavage was performed under surgical microscope. Briefly, the eye was rotated inferiorly by traction suture placed at the limbus, and conjunctival periotomy was performed. A 1.0-mm circumferential incision was made in the globe through the sclera, and into the subretinal space. After placing viscoelastic and cover slip onto the cornea to visualize the fundus, a 30-gauze blunt cannula was carefully inserted through the incision, and placed under the retina. Retina was then gently separated from the RPE by injecting fluid, while avoiding retinal trauma and bleeding. Debridement was performed in approximately 2 × 2 mm^2^ area by gently irrigating BSS into the subretinal space, draining it through the incision site, and rubbing the debris with the needle tip. Upon removal of the debris, all liquid was drained from the subretinal space, and a small amount of balanced salt solution (BSS) was injected into the vitreous cavity to tamponade the detached retina (See Supplementary Movie S1).

### Laser photocoagulation

A 577-nm PASCAL laser (Topcon Medical Laser Systems) was used to deliver patterned laser lesions. Laser power was titrated to produce barely visible retinal burns. The following laser parameters were used: 40 mW power; 15 ms duration; 100 µm spot size in air (45 µm on the retina), 1.5-spot size spacing between spots. Using 5 × 5 gird patterns, retina surrounding the optic disc was irradiated, and the total irradiated retinal area was approximately 1.6 × 1.6 mm^2^ (Fig. 4A).

### Full-field and multifocal electroretinography

For full-field flash ERGs, animals were anesthetized after 12-hour overnight dark adaptation. Pupils were fully dilated with 1% tropicamide and 2.5% phenylephrine solutions, and cornea was lubricated with 1% methylcellulose. A reference/ground needle electrode was placed subcutaneously on the nose. ERG responses were recorded from both eyes simultaneously with the flash intensity of 0.0001, 0.001, 0.01, 0.1, 1, and 3 cd.s/m^2^ using the Dome stimulation with Espion E2 electrophysiology recording system (Diagnosys LLC). At each stimulus intensity, ten flashes of 4 ms were delivered to both eyes simultaneously with 10-second inter-stimulus intervals, and acquired ERG waveforms were averaged. The ERG was recorded at 19, 38, 52, 73, 98, 120, 150, and 180 days of age. For multifocal ERG (mfERG), a binary sequence of black and white squares (20 × 20) was generated using Matlab (The Mathworks, Psychtoolbox) and displayed in front of the animal from a 21-inch liquid crystal display (LCD), with 60 Hz refresh rate. A recording electrode was placed at the corneal limbus. Subcutaneous nose needle electrodes served as reference and ground. Before mfERG recording, the eye was aligned with the center of the LCD monitor (model P2017H, Dell), and placed 12-cm away from it. In the pseudorandom binary m-sequence, the luminance of the squares was either 250 cd/m^2^ (light) or 0.25 cd/m^2^ (dark). One square element was 2.87 cm in width, corresponding to 13.7 deg of the visual angle. Each frame was presented for 100 ms, followed by a black screen for 400 ms (2 Hz image switch). The mfERG signals were analyzed offline with a custom Matlab script.

### OCT imaging

Spectral-domain OCT images were obtained by using HRA2-Spectralis (Heidelberg Engineering), with the cornea covered with viscoelastic and a coverslip to cancel its optical power. Pupils were fully dilated and throughout the procedure, 1% methylcellulose was used to maintain corneal clarity. Surgically treated rats were imaged from P38 through P180 at the same day as ERG recordings. ONL layer thickness was measured at the center of surgically treated area and at corresponding area in the fellow eye. For laser-treated rats, OCT scans were performed from P19 through P180, and thicknesses of the whole retina and ONL layer were measured at five different points, approximately two disc diameters superior to the optic disc. Thickness measured in the treated (right) and control (left) eyes was averaged among all the animals, and plotted as a function of age.

### Histology

For histology, animals treated surgically were sacrificed at P38, P120, and P180, while the laser-treated animals - at P52, P110, and P180. Before enucleation, the superior edge of the eye was marked under deep anesthesia. Both eyes of each animal were enucleated and fixed in 1% paraformaldehyde and 1.25% glutaraldehyde fixative prepared with 5 mM calcium chloride and 5% sucrose for 24 hours at room temperature. The cornea and lens were removed, leaving a posterior eye cup, which was dehydrated through a graded series of alcohols, infiltrated in propylene oxide and embedded in epoxy. The 0.5 µm-thick sections were taken using Reichert Ultracut E, stained with 0.5% toluidine blue, and serial sections of the slides were examined by a light microscope. Images (20x) were taken at a similar area from each sample; at the center of surgical zone or laser-treated zone. The number of photoreceptor nuclei was counted using one image per each eye, and averaged for analysis.

### Transmission Electron Microscopy

The 500 nm-thin epoxy sections were stained with toluidine blue and observed under light microscope for precise localization, before obtaining ultrathin sections. The 70 nm ultrathin sections were collected onto formvar-coated copper grids and dried overnight. Next day sections were stained with uranyl acetate for 30 minutes, washed in PBS, and then stained with lead citrate for 7 minutes. Sections were again washed and dried before observing under transmission electron microscope (JEOL JEM 1400).

### Immunohistochemistry

For immunohistochemistry, animals were sacrificed at P360. All eyes were fixed in 4% paraformaldehyde fixative (Electron Microscopy Sciences, Hatfield, PA, USA) for 24 hours at room temperature. The eyes were cryo-protected in a 30% sucrose solution and 12 μm-thick cryosections were taken using the Leica CM3050 S Cryostat (Wetzler, Germany). The retinal sections were permeabilized in 1% Triton X100 for 3 minutes at room temperature. The sections were blocked for 1 hour in modified PBS with 10% bovine serum albumin (BSA) at room temperature. The tissue was incubated in primary antibody at 4 degrees Celsius overnight in a modified 5% BSA, 0.1% Triton X100, PBS solution. The following primary antibodies were used: rabbit anti-cone arrestin (1:200; Millipore, Billerca, MA, USA), rabbit anti-rhodopsin (1:200; EMD Millipore Co., CA, USA), rabbit anti-IBA1 (1:200; Wako, Richmond, VA, USA), and goat anti-GFAP (1:200; Santa Cruz Biotechnology, Inc., Dallas, TX, USA). After the incubation time and wash steps, the primary antibodies were paired with the appropriate secondary antibodies for 2 hours at room temperature. The following secondary antibodies were used: donkey anti-rabbit Alexa fluor 488 nm (1:200; Thermo Fisher Scientific, CA, USA), donkey anti-rabbit Alexa fluor 568 nm (1:200; Abcam, CA, USA), and donkey anti-goat Alexa fluor 594 nm (1:200; Thermo Fisher Scientific, CA, USA). The sections were mounted with Vectashield DAPI (H-1000; Vector Laboratories, Burlingame, CA, USA). All retinal sections were imaged on a Ziess LSM 880 confocal microscope (Carl Zeiss, Jena, Germany) with x20, x40 or x63 objectives. ImageJ (http://imagej.nih.gov/ij/) was used to generate Z-projections.

### Data analysis

Statistical analyses were performed using the SPSS software (version 24.0, IBM) or Matlab (The Mathworks). Statistical significance of the differences between the treated and control eyes in ONL thickness, b-wave amplitudes, and the number of nuclei in the ONL was determined with a two-sided paired t-test. A p-value < 0.05 was considered statistically significant.

## Electronic supplementary material

Supplementary Information

Surgical removal of subretinal debris
