# Supplementary material for: Long-term Rescue of Photoreceptors in a Rodent Model of Retinitis Pigmentosa Associated with MERTK Mutation
Source: Sci Rep. 2018 Jul 27;8:11312. doi: 10.1038/s41598-018-29631-z (PMC6063887; doi:10.1038/s41598-018-29631-z)
Supplement: Supplementary file 1 — Supplementary Information [file 41598_2018_29631_MOESM1_ESM.pdf]

Supplementary Information:

Long-term Rescue of Photoreceptors in a Rodent Model of Retinitis Pigmentosa Associated with MERTK Mutation

H. Lorach[1*,#], S. Kang[1,2,3*], R. Dalal[2], M.B. Bhuckory[1,2], Y. Quan[2], D. Palanker[1,2]

[1]Hansen Experimental Physics Laboratory, Stanford University, CA, USA

[2]Department of Ophthalmology, Stanford University, CA, USA

[3]Department of Ophthalmology and Visual Science, College of Medicine, The Catholic University of Korea, Seoul, Republic of Korea

[*] These authors contributed equally

[#] Correspondence to henri.lorach@gmail.com

Supplementary Materials

Movie S1. Surgical removal of subretinal debris. This video illustrates the surgical procedure in RCS rats.

Supplementary Figure S1.

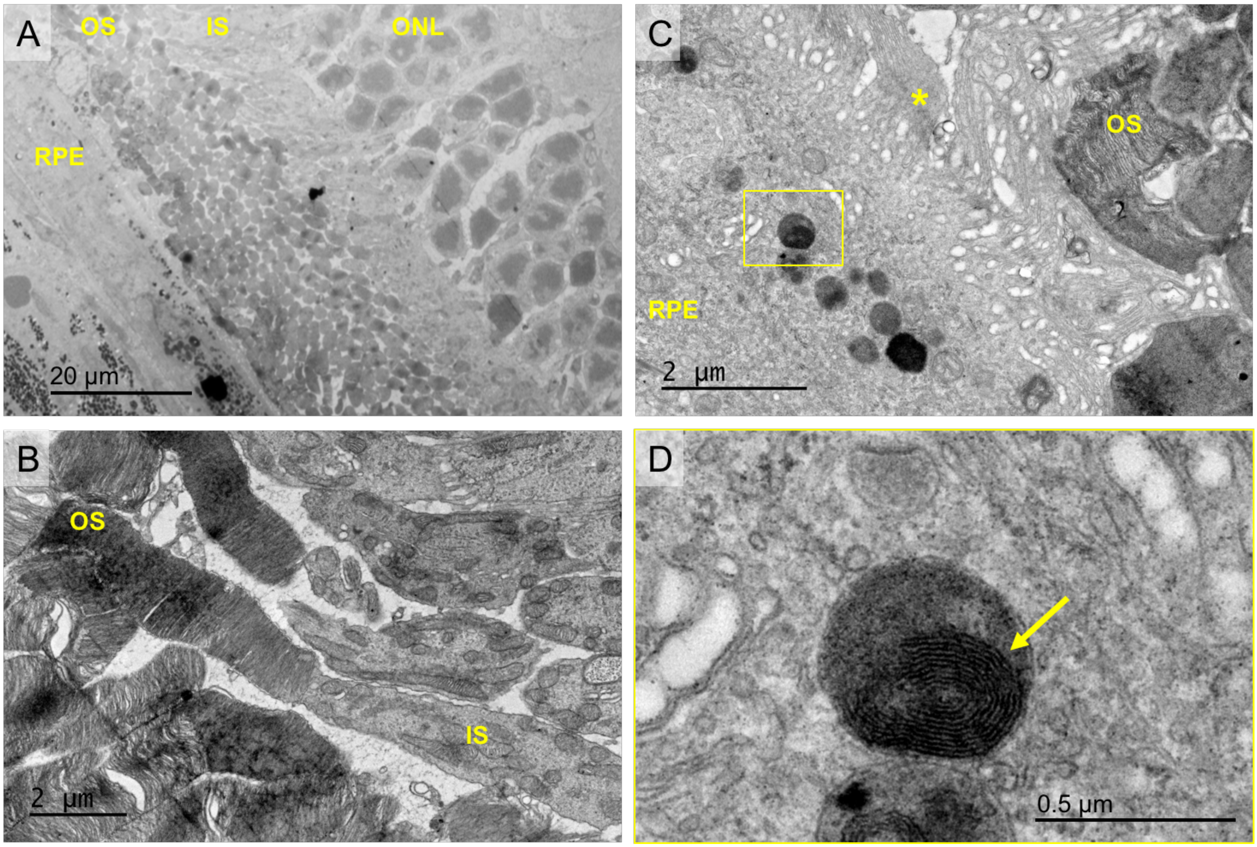

Figure S1: Transmission Electron Microscopy of the surgically-treated area, at P180. A) Overview of the outer retina in the treated zone. B) High magnification of the inner and outer segments revealing the regular disks structure in the outer segments. C) The OS/RPE interface with microvilli (*) as well as phagosomes (within yellow square) containing phagocytized disks (D, arrow).

Supplementary Figure S2.

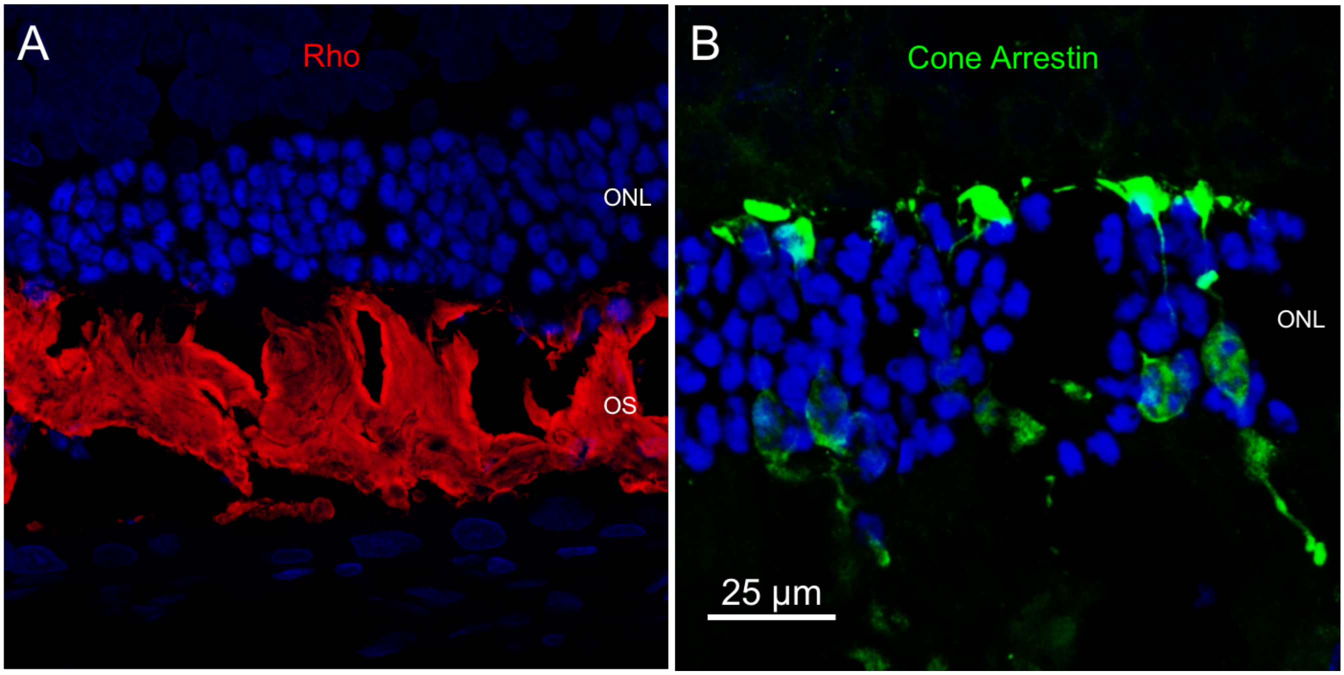

Figure S2. Immunohistological analysis of the surgically treated retina at P360. A) Rhodopsin expression in the outer segments confirms their functional state. (B) Cone arrestin staining shows preservation of cones among the rescued photoreceptors in the rod-dominated rat retina.

Supplementary Figure S3.

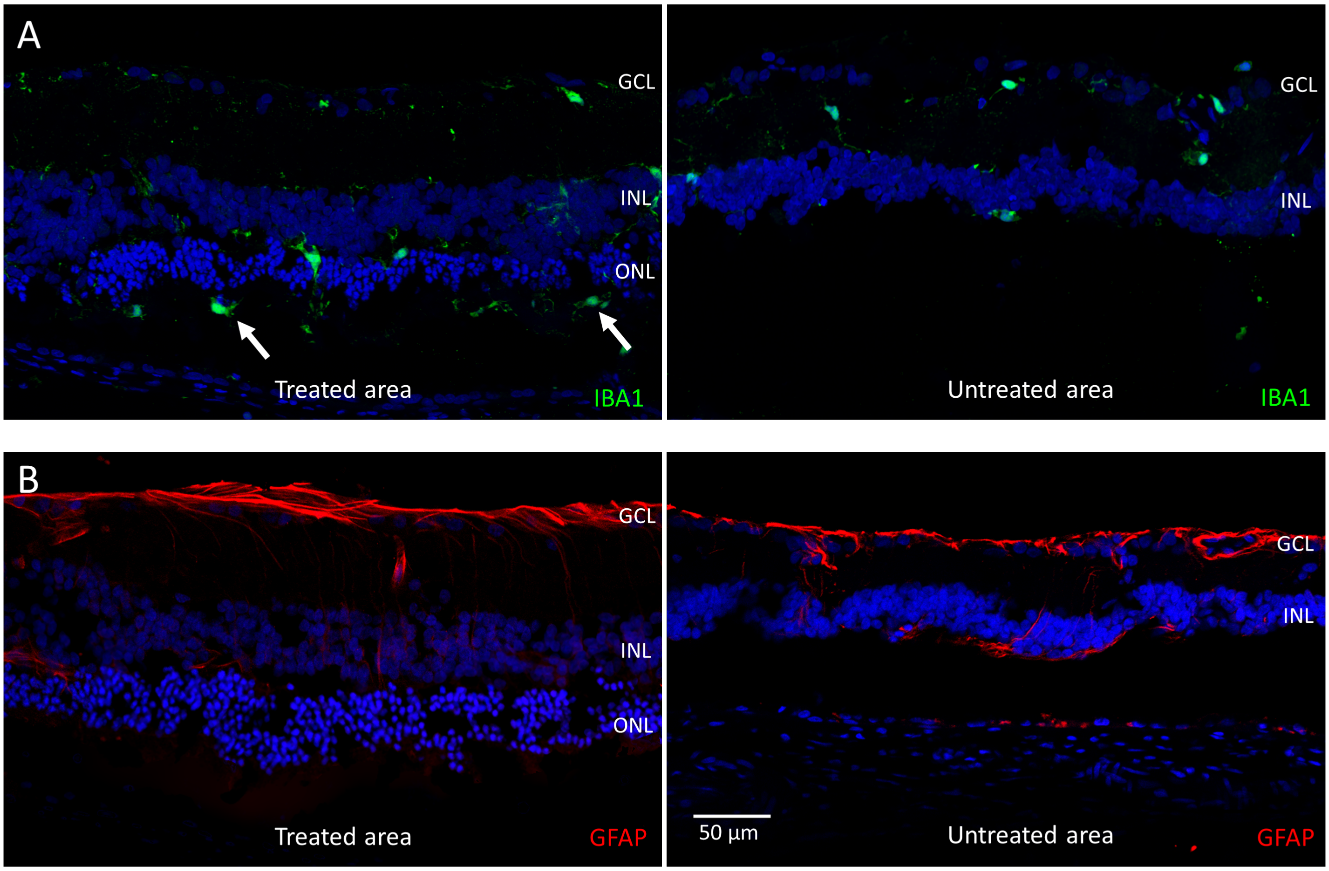

Figure S3. Immunoreactivity of IBA1 (A) and GFAP (B) in the treated eye at P360. Microglial cells (A) are present in the inner and outer retina, including the outer segments area (arrows). Muller cells are activated to a similar extent in treated and untreated areas (B).
